# Determinants and neonatal outcomes of preeclampsia among women living with and without HIV at a tertiary hospital in Zambia: a review of medical records

**DOI:** 10.11604/pamj.2022.43.110.34390

**Published:** 2022-10-28

**Authors:** Moses Mukosha, Bellington Vwalika, Mwansa Ketty Lubeya, Andrew Kumwenda, Patrick Kaonga, Choolwe Jacobs, Kunda Mutesu Kapembwa, Luwi Mercy Mwangu, Patrick Musonda

**Affiliations:** 1 Department of Pharmacy, School of Health Sciences, University of Zambia, Lusaka, Zambia,; 2 HIV and Women's Health Research Group, University Teaching Hospital, Lusaka, Zambia,; 3 Young Emerging Scientists Zambia, Lusaka, Zambia,; 4 Department of Obstetrics and Gynecology, School of Medicine, University of Zambia, Lusaka, Zambia,; 5 Women and Newborn Hospital, University Teaching Hospitals, Lusaka, Zambia,; 6 Department of Epidemiology and Biostatistics, School of Public Health, University of the Witwatersrand, Johannesburg, South Africa,; 7 Department of Neonatology, Women and Newborn Hospital, Lusaka, Zambia

**Keywords:** Preeclampsia, determinants, neonatal outcomes, HIV-infection, antiretroviral therapy, Zambia

## Abstract

**Introduction:**

pre-eclampsia, a pregnancy-specific condition that occurs after 20 weeks of gestation, is a significant public health problem. In the extant literature, there are still conflicting reports on whether Human immunodeficiency virus (HIV) infection and antiretroviral therapy (ART) affect preeclampsia rates. We, therefore, explored the determinants and neonatal outcomes of preeclampsia among pregnant women living with and without HIV.

**Methods:**

we reviewed delivery registers and neonatal files from the 1^st^ January 2018, to 30^th^ of September 2019 for women who delivered at Women and Newborn Hospital. The logistic regression model estimated the odds of preeclampsia and described the neonatal outcomes.

**Results:**

the prevalence of preeclampsia was 7.7% (95% confidence intervals: 6.8 to 8.7). On ART, pregnant women with HIV infection were less likely to develop preeclampsia than those without HIV infection (aOR=0.50; 95% CI: 0.32 to 0.80). However, neonates born to women with preeclampsia were more likely to be admitted to kangaroo mother care than neonates born to normotensive women, regardless of the HIV-exposure status.

**Conclusion:**

overall, the prevalence of preeclampsia was 7.7%, but it was less common among HIV-infected pregnant women receiving ART. Neonates born from women with preeclampsia are at increased risk of adverse outcomes, including admission to kangaroo mother care. These findings underscore the need for healthcare workers to direct their efforts on early diagnosis and detection of preeclampsia in pregnant women to prevent poor outcomes.

## Introduction

Preeclampsia (PE), a pregnancy-specific condition that occurs after 20 weeks of gestation, affects 4-8% of pregnant women globally [1] and 2-13% in sub-Saharan Africa (SSA) [2]. Evidence suggests that PE is among the top five leading causes of morbidity, mortality and poor neonatal outcomes of pregnant women in SSA countries [3]. In Zambia, the prevalence of PE was reported at 12% [4]. Preeclampsia is a new-onset of hypertension (HTN) (systolic BP [SBP] ≥140 mm Hg and diastolic DBP [DBP] ≥90 mm Hg) after 20 weeks of gestation and the presence of significant proteinuria or other maternal organ dysfunction with no evidence of urinary tract infection (UTI) in a random urine sample [5]. Although the aetiology of PE remains unknown, several theories have been proposed [6-8]. The prominent one is that PE develops due to an excessive generalised maternal inflammatory response during pregnancy [9,10]. In addition, it is thought that HIV infection increases the risk of PE through the shared inflammatory process characteristic of the two conditions, but this theory is still controversial among researchers [6,11,12]. Another theory proposes an up-regulation of the immune response at the time of pregnancy, with PE representing an excessive generalised maternal inflammatory response [6,13]. Therefore, immune hyperreactivity may be inhibited when a condition of acquired/induced immune deficiency is present, as in the state induced by HIV infection, which may lower the prevalence of PE [6]. However, the introduction of ART to treat HIV infection appeared to have reversed this effect through immune reconstitution, although no conclusive evidence is available to date [14-20].

With the current HIV epidemic, there has been a concerted effort worldwide to understand the impact of HIV infection and ART on pregnant women [1,21,22,24]. Preeclampsia was the most common adverse outcome among pregnant women with HIV infection on ART in South American and European studies [25,26]. Studies in the Latin American and Caribbean countries combined and Spain found that HIV-infected pregnant women on ART had a five and a two-fold increase in the likelihood of PE compared to HIV-uninfected women, respectively [26,27]. A study in Zambia showed that HIV-infected women on ART had higher odds of hypertensive disorders during pregnancy than those without HIV infection [28]. However, the results have not been consistent; other studies have found no association [6,13,29] or lower risk of PE among HIV-infected women on ART compared to women without HIV infection [11,30]. Others have questioned this association between HIV, ART and PE [13,29,31-33].

Neonates born from women with PE, mainly those born prematurely, are at risk of severe morbidity, mortality, and developmental problems [2], which could, in turn, have long-term effects on overall health during adulthood [34]. Currently, there is no cure for PE [35]. Therefore, early identification of PE (and, if possible, prevention) is a core principle of adequate management [36], and in most cases, the solution is delivery, which should be considered only when the woman has been stabilised [37]. Therefore, it is suggested that women at high risk of PE be identified before week 13 of gestation and low-dose aspirin commenced until 36 weeks' gestation [38]. Even though PE is responsible for increased maternal morbidity and mortality in SSA, the determinants and neonatal outcomes are still poorly documented. Given the differences in ethno-geographic risk factors, the high rate of maternal mortality in SSA, and the uncertainty of whether HIV infection lowers the rate of PE, it is crucial to understand how the two conditions interact. Therefore, the present study had two objectives. The first was to determine socio-demographic, clinical, and/or obstetric determinants of PE among women with and without HIV. The second was to quantify the odds of adverse neonatal outcomes in women with PE compared to those without PE.

## Methods

**Study design, setting and population:** we reviewed medical records at the largest national referral hospital for obstetric and gynaecological conditions, the Women and Newborn Hospital (WNH) in Lusaka, Zambia [2]. On average, the hospital admits about 28,800 pregnant women per year [39]. In addition, the WNH hospital receives referrals from over 20 clinics and five first-level hospitals from areas surrounding Lusaka city and districts from other parts of the country. We included women who delivered at the WNH and whose infants were admitted to the neonatal intensive care unit (NICU) or kangaroo mother care (KMC) between 1^st^ January 2018 and 30^th^ September 2019. In addition, we reviewed neonatal files with a follow-up period of 28 days from admission to assess any adverse outcomes.

**Figure 1:**
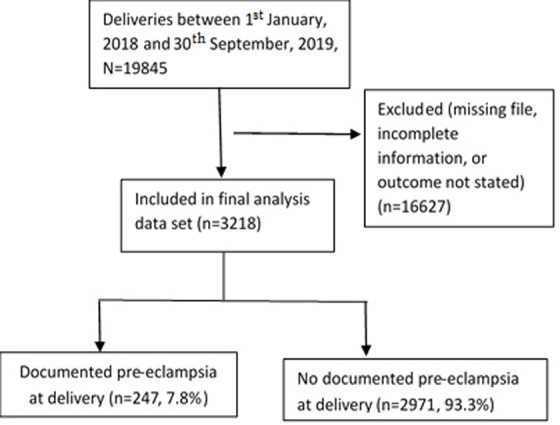
flow of participants in the study; N-population size and n-sample size

**Data extraction:** a complete enumeration of eligible clinical records (delivery notes and neonatal files) was conducted. Records were eligible if they reported information about the women and their neonates delivered at the Women and Newborn hospital. Before further review, we screened all eligible files for completeness of the information (preeclampsia and neonatal clinical outcomes). After the screening process, de facto eligible sample came to 3218 records (Figure 1). A predesigned data entry form was used to capture data. In addition, relevant socio-demographic, obstetric, neonatal and clinical data were extracted from the delivery records and neonatal files. Demographic, clinical and obstetric data included antenatal care (ANC) attendance, maternal age, HIV status, gravidity, parity, history of preterm birth, substance use, 28-day neonatal mortality, respiratory distress syndrome and admission to KMC. Details of the data auditing and cleaning have previously been reported [40].

**Study variables:** the primary outcome of interest for the first objective was PE measured on a binary scale (coded, yes=1, no=0). In addition, the following information was obtained from records; admission to KMC/28-day mortality/development of respiratory distress syndrome (RDS) within 28 days of admission (coded, yes=1, no=0), gravidity (i.e. the number of previous pregnancies, including the present one and miscarriages and ectopic pregnancies [41,42]); maternal age (number of years from last birthday); parity (number of times a woman has given birth to a live fetus with gestational age above 28 weeks) [43]; HIV serostatus (positive/ negative); employment status (yes, no); ANC attendance (yes, no), never used any substance (i.e. alcohol or smoking) and history of preterm delivery (yes, no). Diagnosis of PE in the study setting follows the ACOG criteria defined as a new-onset of HTN (SBP ≥140 mm Hg and diastolic DBP ≥90 mm Hg) after 20 weeks of gestation and the presence of proteinuria or other maternal organ dysfunction with no evidence of UTI in a random urine sample [5].

**Statistical analysis:** we used Stata/IC version 16.1 (Stata Corp., College Station, Texas, USA) for statistical analysis. Descriptive analysis was done on participants' socio-demographic, obstetric, and clinical characteristics. For the descriptive table, parity, age and gravidity were categorised. We reported categorical variables as frequencies and percentages. Test of associations was done using the Pearson Chi-square test or Fisher's exact test as appropriate. For the first objective, predictors of PE were investigated in both univariable and multivariable analyses using logistic regressions. Variables for inclusion in the multivariable model were selected based on the liberal cut of p<0.2 from the univariable models. Crude and adjusted odds ratios with accompanying 95% confidence intervals were calculated.

For the second objective, women with PE and those without were compared and crude and adjusted odds were estimated for three adverse neonatal outcomes; admission to kangaroo mother care, neonatal mortality (death within 28 days) and respiratory distress syndrome (within 28 days of admission). In the multivariable analysis, each outcome model was adjusted for the multivariable predictors model (HIV-serostatus, gravidity, parity, employment status and maternal age). Once again, the logistic regression model approach was adopted, and odds ratios were calculated with accompanying 95% confidence intervals. We considered age, parity, and gravidity as continuous variables for all the regression models to avoid inflating the type I error rate. Interactions between significant variables were investigated, and none was found to approach statistical significance. Multiple imputations were done to assess whether the missing data for age, gravidity, parity and employment, if imputed, could affect the association observed between HIV-serostatus and preeclampsia. The missing data did not affect the estimates, so a complete case analysis was performed. We used Hosmer-Lemeshow goodness-of-fit test to assess the models' predictive ability. All statistical tests were done at a 5% significance level and 95% confidence intervals.

**Ethical considerations:** we submitted the protocol to the University of Zambia Biomedical Research Ethics Committee (UNZABREC) for approval; reference number UNZA-221/2019. Additional permission was obtained from National Health Research Authority and the Women and Newborn Hospital management to extract data. This study used medical records from delivery notes and neonatal files. There was no direct contact with participants; therefore, no informed consent was obtained. In addition, we de-identified data to protect the participant's confidentiality.

## Results

Table 1 shows study participants' demographic, obstetric, and clinical characteristics. From the total 3218, the majority, 1257 (41.8%), were in the age group 25-34 years, 1427(48.2%) had been pregnant more than twice, and 2134 (72.0%) had given birth 1-3 times before. In addition, about one-fifth, 640 (19.9%) were living with HIV and 2395 (93.3%) were employed. On the other hand, nearly everyone, 3155 (98.0%) had attended ANC at least once, 3211 (99.9%) never used any substance (i.e. alcohol or smoking) during pregnancy, and 3194 (99.5%) had no history of preterm birth. Overall, 247/3218 developed PE during the data collection period; this translates to a prevalence of 7.7% (95% confidence intervals [CI]: 6.8 to 8.6). When stratified by HIV status, the prevalence of PE among women living with HIV was 4.8% (95% CI: 3.3 to 6.8) and 8.4% (95% CI: 7.3 to 9.5) among HIV uninfected women. In addition, there was evidence of a difference between women with and without PE on the following: maternal age (p<0.001), HIV-serostatus (p=0.003), and employment status (p<0.001). Table 2 shows the prevalence of PE by HIV-serostatus. For the HIV negative pregnant women, the prevalence was highest in the age group 25-34 years, 102/213 (47.9%), among women who had been pregnant more than twice, 103/216 (48.4%), had parity between 2-4, 106/205 (51.7%) and were unemployed, 150/216 (87.4%). On the other hand, among women living with HIV, the prevalence was highest among women in the age group 25-34 years, 16/30 (53.3%), who had been pregnant at least twice, 17/31 (54.8%), had parity between 2-4, 16/28 (57.1%), and were unemployed, 21/31 (87.5%).

**Table 1:** demographic, obstetric, and clinical characteristics of women admitted to women and new born hospital by preeclampsia status

Characteristic	Level	Total population N=3218 (%)	Preeclampsia		P-value
			Yes, n=247	No, n=2971	
HIV serostatus	Negative	2578(80.1)	216(87.5)	2362(79.5)	0.003^a^
	Positive	640(19.9)	31(12.6)	609(20.5)	
Age (years)	14-24	1216(40.4)	59(24.3)	1157(41.9)	<0.001^a^
	25-34	1257(41.8)	118(48.6)	1139(41.2)	
	≥35	534(17.8)	66(27.2)	468(16.9)	
Parity	1	1121(37.8)	75(32.2)	1046(38.3)	0.180^a^
	2-4	1427(48.2)	122(52.4)	1305(47.8)	
	≥5	415(14.0)	36(15.5)	379(13.8)	
Gravidity	1	1036(34.5)	74(30.3)	962(34.8)	0.183^a^
	2	658(21.9)	50(20.5)	608(22.0)	
	> 2	1313(43.7)	120(49.2)	1193(43.2)	
Employment status	Unemployed	2395(93.3)	171(87.2)	2224(93.8)	<0.001^a^
	Employed	173(6.7)	25(12.8)	148(6.2)	
ANC attendance	No	63(1.9)	3(1.2)	60(2.0)	0.629^b^
	Yes	3155(98.0)	244(98.8)	2911(97.9)	
Substance use (alcohol, smoking)	No	3211(99.9)	246(99.6)	2965(99.9)	0.213^b^
	Yes	3(0.1)	1(0.4)	2(0.07)	
History of preterm birth	No	3194(99.5)	247(100)	2947(99.5)	0.629^b^
	yes	16(0.5)	-	16(0.5)	

a Pearson Chi-square test; ^b^Fischer's exact test; PE-pre-eclampsia; ANC-antenatal care; HIV-human immunodeficiency virus; all percentages are reported on complete cases

**Table 2:** preeclampsia prevalence by HIV-serostatus among women admitted to Women and Newborn Hospital

Characteristic	Level	HIV seronegative N=216 (87.5%)		HIV seropositive N=31 (12.6%)	
		n (%)	95% CI	n (%)	95% CI
Age (years)	14-24	50(23.5)	17.9-29.7	9(30.0)	14.7-49.4
	25-34	102(47.9)	41.0-54.8	16(53.3)	34.3-71.7
	≥35	61(28.6)	22.7-35.2	5(16.7)	5.6-34.7
Parity	1	67(32.7)	26.3-39.6	8(28.6)	13.2-48.7
	2-4	106(51.7)	44.6-58.7	16(57.1)	37.2-75.5
	≥5	32(15.6)	10.9-21.3	4(14.3)	4.0-32.0
Gravidity	1	65(30.5)	24.4-37.2	9(29.0)	14.2-48.0
	2	45(21.1)	15.9-27.2	5(16.1)	5.5-33.7
	> 2	103(48.4)	41.5-55.3	17(54.8)	36.0-72.7
Employment status	Unemployed	150(87.2)	81.3-91.8	21(87.5)	67.6-97.3
	Employed	22(12.8)	8.2-18.7	3(12.5)	2.7-32.4
ANC attendance	No	2(0.9)	0.1-3.3	1(3.2)	0.1-16.7
	Yes	214(99.1)	96.7-99.9	30(96.8)	83.3-99.9
Substance use (alcohol, smoking)	No	215(99.5)	97.5-99.9	31(100)	88.8-100*
	Yes	1(0.5)	0.1-2.6	-	-

Key: *=one-sided; 97.5% confidence interval; ANC-antenatal care; PE-pre-eclampsia; HIV- human immunodeficiency virus

**Maternal factors associated with preeclampsia:** the univariable and multivariable logistic regression models are shown in Table 3. Pregnant women with HIV infection on ART compared to those without HIV infection had 50% reduced odds of PE (aOR 0.50; 95% CI 0.32 - 0.80; p=0.004). We note that the clinical, obstetric and socio-demographic factors associated with PE were maternal age (aOR 1.06; 95% CI 1.04-1.10; p<0.001), parity (aOR 0.76; 95% CI 0.62 - 0.92; p=0.006), gravidity (aOR 1.25; 95% CI 1.02 - 1.53; p=0.028) and employment status (aOR 1.92; 95% CI 1.20 - 3.07; p=0.005). A unit increase in age and gravidity was associated with higher odds of PE (6% and 25%, respectively). Similarly, women in employment had 92% higher odds of PE than those unemployed. Conversely, a unit increase in parity was associated with a 24% reduced odds of PE. In the multivariable analysis of neonatal outcomes associated with PE, the adjusted odds of admission to KMC were 85% higher for neonates born to preeclamptic women than those born to normotensive women regardless of HIV-exposure status. However, there was no evidence of PE increasing the odds of neonatal mortality within 28 days and RDS development among the neonates (Table 4).

**Table 3:** univariable and multivariable logistic regression model of maternal factors associated with preeclampsia

Characteristic	Level	Crude OR (95% CI)^b^	P-value	Adjusted OR (95% CI)^c^	P-value
HIV sero-status	Negative	Ref	0.003	Ref	0.004
	Positive	0.56(0.38-0.82)		0.50(0.32-0.80)	
Age (years)	A unit increase	1.05(1.03-1.07)	<0.001	1.06(1.04-1.10)	<0.001
Parity	A unit increase	1.05(0.98-1.13)	0.167	0.76(0.62-0.92)	0.006
Gravidity	A unit increase	1.08(1.01-1.16)	0.030	1.25(1.02-1.53)	0.028
Employment status	Unemployed	Ref	0.001	Ref	0.005
	Employed	2.20(1.40-3.45)		1.92(1.20-3.07)	

Key: OR-odds ratio; 95% CI- 95% confidence intervals; HIV-human immunodeficiency virus; amissing values were excluded from the analysis; ^b^Calculated from univariable logistic regression of the effect of each factor on odds of preeclampsia. ^c^Calculated from multivariable logistic regression of the combined effect of all factors on preeclampsia. The sample size for the complete-case analysis multivariable regression 2,351.

**Table 4:** neonatal outcomes of newborns from preeclamptic women admitted to women and new born hospital

Outcomes	PE/no N(%)	PE/yes N(%)	Crude OR (95% CI)^a^	P-value	Adjusted OR (95% CI)^b^	P-value
**Mortality within 28 days (model 1)**						0.751
No	1163(39.15)	105(42.51)	Ref	0.299	Ref	
Yes	61808(60.85)	142(57.49)	0.87(0.67-1.13)		0.95(0.70-1.30)	
**Admission to KMC (model 2)**						<0.001
No	1965 (66.23)	138(55.87)	Ref		Ref	
Yes	1002(33.77)	109(44.13)	1.55(1.19-2.01)	0.001	1.85(1.35-2.53)	
**RDS (model 3)**						0.968
No	1607(54.14)	123(49.80)	Ref		Ref	
Yes	1361(45.86)	124(50.20)	1.19(0.92-1.54)	0.188	0.99(0.73-1.35)	

Key: KMC-kangaroo mother care; OR-odds ratio; 95% CI- 95% confidence intervals; ^a^Calculated from univariable logistic regression of the effect of preeclampsia on each of the outcomes; ^b^Calculated from multivariable logistic regression of the effect of preeclampsia on each of the outcomes; adjusting for all risk factors (age, parity, gravidity, employment and HIV) shown in table 3; cunless otherwise stated all values are frequencies (percentages); dmissing values excluded; gravidity n=211; parity n=255; employment status n=650

## Discussion

The present study aimed to assess determinants of PE and the effect of the condition on neonatal outcomes at a national referral hospital in Lusaka, Zambia. Over the study period, we found the overall prevalence of 7.7%, being significantly higher among HIV-uninfected pregnant women, 8.4%, compared to HIV-infected 4.8%. Multivariable logistic regression showed the reductive effect of HIV infection and ART on PE. Among the factors associated with PE were, maternal age, gravidity parity and employment status. The odds of PE increased as age and gravidity increased and the odds decreased as parity increased. Employment compared to unemployment increased the odds of PE. In addition, PE was associated with increased odds of admission to KMC for neonates. In a secondary analysis, the odds of admission to KMC nearly doubled for the neonates born to women with PE comorbid HIV infection on ART.

The prevalence we found is consistent with global estimates of between 4% to 8% [1] and 2% to 11% in SSA [2]. However, the prevalence of PE from our study is lower than those reported in Ethiopia of 8.4% to 12.4%, in South Africa 12.5% [33,44], Zimbabwe 10.4% [45], and in Zambia 5 years ago of 12.5% [4]. Nevertheless, this prevalence was higher than that reported in Tanzania 3.3% [46], Nigeria 1.2% [47], and the Democratic Republic of Congo 4.8% [48]. Further, a clinical audit conducted at the Women and Newborn Hospital from 1^st^ January 2020 to 31^st^ July 2021 found a point prevalence of severe PE of 12% among patients admitted to the special observation unit [49]. The wide variability in the prevalence of PE could be partly related to differences in the risk factors and predictors of PE in different parts of the world and methodological differences [50-52]. For instance, large numbers of study participants, 3,218 were included in our study, but only 129 and 1093 study participants were included in the study from Ethiopia and South Africa, respectively. Additionally, our population was restricted to women whose infants were admitted to NICU and KMC. Moreover, the etiology of PE remains unknown, making the comparisons across jurisdictions difficult.

Recently, HIV infection and ART use have been implicated in the increase in PE prevalence, despite inconsistent results from several studies [26-28,33]. In the present study, the prevalence of PE among the HIV-infected group was consistent with reported estimates elsewhere of between 0.9% to 5% [33,53]. If the postulations regarding the role the immune system plays in the aetiology of PE are correct, then the argument that PE development is partially prevented under conditions of relative immune deficiency is plausible. Wimalasundera and colleagues found that women with HIV infection and not on ART had a significantly lower rate of PE than HIV-infected women on ART [6]. On the contrary, a study in South Africa compared untreated HIV-infected pregnant women to HIV uninfected women and found no reduction in the risk of PE [54].

It follows from the immune deficiency argument that direct or surrogate markers of immune competence, such as the CD4 lymphocyte count or viral load, would better explain differences in the prevalence of an immunologically mediated disease. A study in South Africa found that the prevalence of PE in HIV-infected women did not differ around the CD4 count threshold of 350 cells/mm3 [55]. However, this was contrary to a study in Botswana that reported that prior to ART, HIV-infected women with a high viral load (>100,000 copies) were more likely to develop PE compared to their counterparts with less viral load [24]. The differences probably could be due to the different thresholds of CD4 cell count used in the two studies, multisystemic aetiology of PE and disease stage. ART may increase the risk of PE to a level higher than the background prevalence through its toxic effects that may mimic preeclampsia [27,33]. In the present study, CD4 count and viral load information were missing in delivery notes; therefore, we could not adjust for these variables.

Several determinants of PE were identified in the present study, consistent with the published literature [1, 56-58]. The extant literature suggests that advanced maternal age is a risk factor for developing PE [59]. For instance, a study conducted in Ethiopia showed that maternal age over 35 years was associated with an increased risk of developing PE [60]. In our study, 15% of preeclamptic women were over 40 years old, suggesting that advanced maternal age could be among the risk factors for developing PE in this setting. Furthermore, the overall mean maternal age was 27.2 years in our study, and only 5% of pregnant women were over 40 years old; the majority of pregnant women were under 30 years old, suggesting that lower maternal age could be one of the reasons for the low prevalence of PE in our study. These findings were supported by results from South Africa [53], Iran [58], Germany [61] and Taiwan [62]. These findings could be explained by the fact that as age increases, the chronic elevations in inflammatory mediators during late life contribute to a deleterious chronic overproduction of reactive oxygen species leading to damage of cellular proteins and organelles [63]. This chronic cellular damage and dysfunction are thought to partially contribute to the endothelium's physiologic dysfunction and subsequent hypertension [64,65].

Furthermore, we noted that with the increase in the number of times a woman has been pregnant before, the risk of PE increased by 1.25-fold. On the other hand, PE risk was reduced by 0.76-fold as the number of times a woman has given birth to a live foetus with gestational age above 28 weeks increased. This was corroborated by Maeda *et al*. [66], who reported that multiparity was significantly associated with a low risk of PE. In the present study, women in employment were more likely to develop PE than those not in employment. Few studies have explored this relationship [67]. One plausible explanation could be the differences in work-related stress, social-economic status, physical activity and diet. In addition, women in employment are more likely to experience depression, stress and anxiety, which has previously been linked to hypertension [68].

Among neonatal outcomes, PE was associated with higher odds of admission to KMC. Admission to KMC has been reported to be an effective way to reduce neonatal mortality due to continuous skin-to-skin contact between mother and baby, which improves baby's temperature, support exclusive breastfeeding and helps early recognition/ response to illness [3]. This study has some limitations. Firstly, all HIV-infected women in our study setting receive ART as a routine standard of care. Our study, nonetheless, was not able to quantify the type and time on ART which could have enabled us to assess the independent effect of HIV infection. Despite not having ART adherence data, a pilot study which we conducted at the first-level hospital in Lusaka, Zambia, showed 96% adherence levels among pregnant women [69]. Therefore, we can only postulate to the current setting. Secondly, we could not control for some maternal variables like antenatal prophylaxis with steroids (to reduce the severity of neonatal RDS), prolonged rupture of membranes, diseases during pregnancy, type of pregnancies (multiple or singleton gestation), place of delivery, maternal fever, meconium-stained amniotic fluid and premature rupture of membranes as these were not consistently documented in the delivery records.

## Conclusion

Overall, 7.7% of pregnant women developed preeclampsia, and their neonates had markedly increased odds for adverse outcomes, including admission to kangaroo mother care. Advanced maternal age, increased gravidity, and employment substantially increased preeclampsia odds. The evidence from this study supports the findings that PE is less common in HIV-infected on treatment compared to HIV-uninfected women. However, neonates born to women with PE, regardless of HIV-exposure status, are more likely to develop adverse outcomes. Taken together, these findings underscore the need for early and comprehensive prenatal care. In addition, there is urgent need for expansion of monitoring and timely diagnosis of PE among HIV-infected women on ART given the recent policy changes in Zambia and SSA, where every pregnant woman is commenced on ART regardless of the CD4 cell count.

### What is known about this topic

- Preeclampsia is a significant global public health problem;
- Preeclampsia is responsible for increased maternal morbidity, and mortality;
- Sub-Saharan Africa shares the burden of preeclampsia disproportionately than similar settings.

### What this study adds

- Determinants of preeclampsia in the context of high HIV prevalence;
- Prevalence of preeclampsia at the country’s largest referral hospital and that preeclampsia is less common among women living with HIV infection and on ART;
- Preeclampsia increases the risk of admission to kangaroo mother care.
